# Celastrol Attenuates Multiple Sclerosis and Optic Neuritis in an Experimental Autoimmune Encephalomyelitis Model

**DOI:** 10.3389/fphar.2017.00044

**Published:** 2017-02-10

**Authors:** Hongbin Yang, Chang Liu, Jie Jiang, Yuena Wang, Xiaoyu Zhang

**Affiliations:** ^1^Department of Ophthalmology, The First Affiliated Hospital of Harbin Medical UniversityHarbin, China; ^2^Department of Neurology, Harbin Fourth HospitalHarbin, China; ^3^Department of Neurosurgery, The Second Affiliated Hospital of Harbin Medical UniversityHarbin, China

**Keywords:** multiple sclerosis, optic neuritis, celastrol, inflammation, apoptosis

## Abstract

This study was aimed to evaluate the effects of celastrol, a natural compound with multiple bioactivities, on multiple sclerosis and optic neuritis (ON) in rat experimental autoimmune encephalomyelitis (EAE). EAE was induced in Sprague Dawley rats using myelin basic protein, and the animals received daily intraperitoneal injections of celastrol or vehicle for 13 days. The EAE rats showed abnormal neurobehavior and inflammatory infiltration and demyelination in the spinal cord. Significantly upregulated mRNA expression of pro-inflammatory cytokines interferon-γ and interleukin-17 and downregulated anti-inflammatory cytokines interleukin-4 were found in the spinal cord of EAE rats. In the study of ON, severely inflammatory responses like in the spinal cord were also seen in the optic nerve, as well as obvious microgliosis. Furthermore, activation of nuclear factor kappa-B and upregulated inducible nitric oxide synthase was observed in the optic nerve. In addition, apoptosis of retinal ganglion cells and dysregulation of apoptotic-associated proteins in the optic nerve were found in EAE rats. Treatment of celastrol potently restored these changes. In most of the indexes, the effects of high dose of celastrol were better than the low dose. Our data conclude that administration of celastrol attenuates multiple sclerosis and ON in EAE via anti-inflammatory and anti-apoptotic effects. These findings provide new pre-clinical evidence for the use of celastrol in treatment of multiple sclerosis.

## Introduction

Multiple sclerosis (MS) is a chronic inflammatory and neurodegenerative diseases characterized by demyelination in the central nervous system (CNS; Compston and Coles, 2002). MS is a major cause of neurological dysfunction among young adults worldwide (Bashir and Whitaker, 2002). Until now, the etiology of MS is not completely revealed, but T- and B-cells mediated immune attack is considered to contribute to neuronal damage in the CNS (Lucchinetti et al., 2000; Compston and Coles, 2002). Optic neuritis (ON), inflammation of optic nerve, usually refers to the acute optic neuropathy that results from focal inflammation associated with demyelination (Soderstrom, 2001). ON is the early diagnostic sign in approximately 20% of MS patients, and about 70% patients develop ON during the course of MS (Sorensen et al., 1999; Toosy et al., 2014; Graham and Klistorner, 2016). In addition, most of patients with monosymptomatic ON eventually develop clinically definite MS, and the morbidity increases with years [reviewed in (Soderstrom, 2001)]. In clinical routines, short-term administration of high dose of corticosteroids is used for relieving acute or subacute MS and ON, but long-term use of corticosteroids is not recommended for its adverse effects and much less impact on recovery. Other Food and Drug Administration (FDA)-approved drugs such as interferon, Natalizumab, Mitoxantrone, etc., also have limitation in clinical use because of their serious side effects (Kleinschmidt-DeMasters and Tyler, 2005; Marriott et al., 2010). Therefore, to develop novel compounds those target the clinical disease process including ON is necessary.

Experimental autoimmune encephalomyelitis (EAE) is the most commonly used animal model of MS (Wekerle et al., 1994). This model can mimic several symptoms of the MS in human, such as the inflammation induced by T- and B-cells, demyelination, and optic nerve damage (Storch et al., 1998; Meyer et al., 2001; Das et al., 2013). Apoptotic cell death of retinal ganglion cells (RGCs) occurs early in the process of EAE (Meyer et al., 2001; Fairless et al., 2012). Myelin proteins such as myelin oligodendrocyte protein (MOG), myelin basic protein (MBP) and proteolipid protein are used for EAE model establishment in rodents. In the present study, Sprague Dawley (SD) rats were immunized by MBP and the neurological function and damages to spinal cord and optic nerve were observed.

Celastrol is a quinone methide triterpenoid extracted from traditional Chinese medicine *Celastrus orbiculatus, C. aculeatus, Tripterygium wilfordii* (Thunder God Vine) and other plants of the Celastraceae family (Venkatesha and Moudgil, 2016). Numerous studies demonstrated the pharmacological effects of celastrol on various diseases, including autoimmune diseases, chronic inflammation, neurodegenerative diseases, and many types of cancer (Allison et al., 2001; Salminen et al., 2010; Kannaiyan et al., 2011). Specifically, celastrol showed prominent effects in inflammation control and immunosuppression. Celastrol has been demonstrated to alleviate arthritis in various animal models through regulating the production of pro-inflammatory cytokines and the function of immune cells (Venkatesha et al., 2011; Cascao et al., 2012; Astry et al., 2015). In China, *T. wilfordii* tablet is approved by China Food and Drug Administration (CFDA) for rheumatoid arthritis. Recently studies on EAE animals reported that celastrol may have ability to attenuate MS (Abdin and Hasby, 2014; Wang et al., 2015). In these studies, celastrol was found to regulate Th17 responses, balance the pro- and anti-inflammatory cytokines via modulating Th1 and Th2 responses, and downregulate nuclear factor kappa-B (NF-κB) expression. In the present study, the effect of celastrol on MS was evaluated in EAE rats. Besides the neuronal function and inflammatory responses in spinal cord, inflammation in optic nerve and RGC damage were tested as well.

## Materials and Methods

### Animals

Male SD rats (8–10 weeks, 180∼200 g, the Experimental Animal Centre of Harbin Medical University, Harbin, China) were maintained under a 12-h light/dark cycle with free access to water and food. All animal procedures were approved by the Ethics Committee of Harbin.

### EAE Induction and Celastrol Administration

**FIGURE 1:**
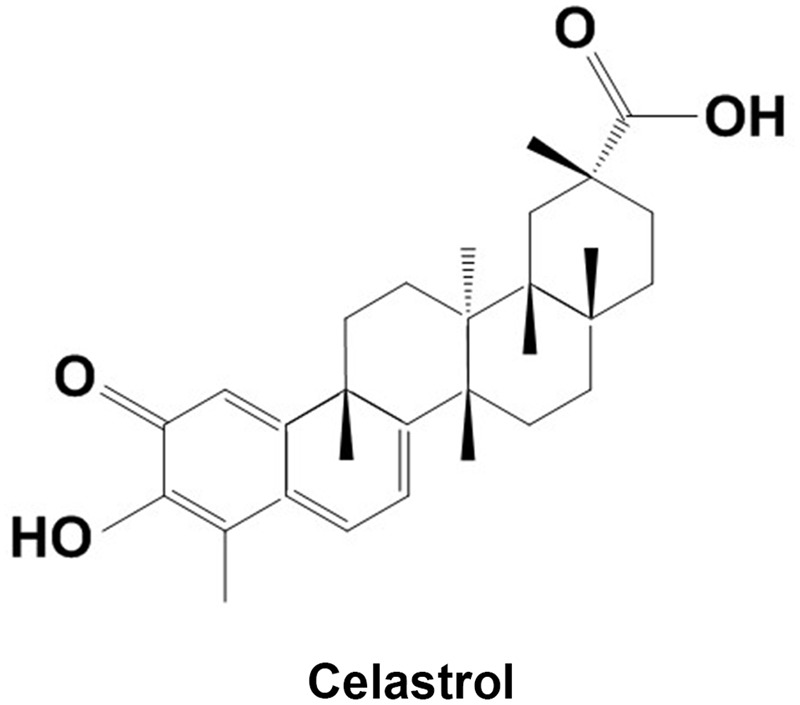
**Chemical structure of celastrol**.

Rats were randomly divided into four groups: (1) control; (2) EAE; (3) EAE + celastrol 1 mg/kg; and (4) EAE + celastrol 2 mg/kg. EAE were induced in the rats by immunization with 50 μg MBP (GL Biochem Ltd., Shanghai, China) and 1 mg/ml mycobacterium tuberculosis emulsified in 100 μL complete Freund’s adjuvant (CFA). Rats in the control group received equal amount of vehicle. Rats in the celastrol groups were intraperitoneally injected daily with indicated dose of celastrol (**Figure 1**, Aladdin, Shanghai, China) for 13 days. Control and EAE rats received the same amount of 1% dimethyl sulfoxide (DMSO). Neurological sign was monitored daily and was scored according to the following scale: 0, no clinical signs; 1, loss of tail tone (limp tail); 2, waddling gait with tail weakness (ataxia); 3, moderate hindlimb paralysis; 4, tetraparesis; and 5, moribund stage. All the rats were sacrificed at day 14 and the spinal cord tissue in C4-T1 vertebra and optic nerve were collected.

### Histological Examination

The spinal cord tissues were fixed in 4% paraformaldehyde for 24 h, embedded in paraffin and cut into 5-μm thickness sections. Haematoxylin and eosin (H&E) staining were used to evaluate the inflammatory cell infiltration and pathological changes in spinal cords. Luxol fast blue (LFB) staining was used to examine demyelination. After being cleared with xylene and hydrated in graded ethanol, the sections were stained with haematoxylin and eosin or LFB (Solarbio Science & Technology, Beijing, China) using standard protocols. The histological changes were observed under a light microscopy (DP73; Olympus, Tokyo, Japan). Histopathological changes were scored by two independent investigators according to the standards used in previous study (Liu et al., 2014) and the averages were used as final scores.

### Terminal Deoxynucleotidyl Transferase-Mediated dUTP Nick End Labeling (TUNEL)

The optic nerves were fixed in 4% paraformaldehyde for 24 h, embedded in paraffin and cut into 5-μm thickness sections. The sections were dewaxed and stained using Terminal Deoxynucleotidyl Transferase-Mediated dUTP Nick End Labeling (TUNEL kit; Roche Diagnostics, Mannheim, Germany) following the manufacturer’s protocol. Sections were counterstained with hematoxylin and observed under the light microscope (DP73, Olympus, Tokyo, Japan).

### Immunofluorescence Staining

Dewaxed optic nerve sections were boiled in 0.1 M sodium citrate buffer for 10 min using a microwave oven to perform the antigen retrieval. The sections were then blocked in goat serum for 30 min at room temperature and incubated in primary anti-CD11b antibody (1:100, ab1211, Abcam, Cambridge, UK) at 4°C overnight. After being washed in PBS, Cy3- conjugated secondary antibodies (goat anti-mouse, 1:200; Beyotime) were applied for 60 min at room temperature in darkness. Fluorescent labeling was visualized under a fluorescence microscope (BX53, Olympus, Tokyo, Japan).

### RNA Isolation and Quantitative Real-Time PCR

Total RNA from the spinal cord tissue and optic nerve was isolated using a RNA simple Total RNA Kit (Tiangen, Beijing, China) according to the manufacturer’s protocol. Complementary DNA was reverse-transcribed from the total RNA from each sample and oligonucleotide primer using super M-MLV (BioTeke, Beijing, China). Quantitative real-time PCR reactions were performed on the synthesized cDNA using 2 × Power Taq PCR Master Mix (BioTeke) and SYBR Green (Solarbio Science & Technology) on an Exicycler 96 real-time quantitative thermal block (Bioneer, Daejeon, Korea). Results were analyzed using 2^-ΔΔCt^ method and represented as fold changes of β-actin. The primers used in the study are listed as follows: interferon-γ (INF-γ), forward: 5′-GCCCTCTCTGGCTGTTACT-3′, reverse: 5′-TTTGCCAGTTCCTCCAGATA; interleukin (IL)-17, forward: 5′-CTACCTCAACCGTTCCACT-3′, reverse: 5′-CTTCTCAGGCTCCCTCTTC-3′; IL-4, forward: 5′-TGATGTACCTCCGTGCTTGA-3′, reverse: 5′-ATTTCCCTCGTAGGATGCTT-3′; IL-5, forward: 5′-AGGCTTCCTGTTCCTACTC-3′, reverse: 5′-TCCATTGCCCACTCTGTA-3′; TNF-α, forward: 5′-TGGCGTGTTCATCCGTTCT-3′, reverse: 5′-CCACTACTTCAGCGTCTCGT-3′; IL-1β, forward: 5′-TCCAGTCAGGCTTCCTTGTG-3′, reverse: 5′-CGAGATGCTGCTGTGAGATT-3′; inducible nitric oxide synthase (iNOS), forward: 5′-CTAGACCTCAACAAAGCTCTC-3′, reverse: 5′-GAAGAACAATCCACAACTCGC-3′; β-actin, forward: 5′-GGAGATTACTGCCCTGGCTCCTAGC-3′, reverse: 5′-GGCCGGACTCATCGTACTCCTGCTT-3′.

### Western Blotting Analysis

The spinal cord tissues and optic nerves were homogenized in cooled RIPA buffer supplement with phenylmethanesulfonyl fluoride (Beyotime) on ice. The homogenate was centrifuged at 15,000 *g* for 10 min at 4°C and the supernatants were collected. Nuclear and cytosolic proteins were extracted using a Nuclear and Cytoplasmic Protein Extraction Kit (Beyotime) according to the manufacturer’s protocol. Concentration of the protein was determined using a Bicinchoninic Acid (BCA) Protein Assay Kit (Beyotime). Equal amount of proteins in each sample was separated on 10% SDS-PAGE and transferred onto polyvinylidene fluoride membranes (Millipore, Billerica, MA, USA). After being blocked with 5% non-fat dry milk (w/v), the membranes were incubated in primary antibodies at 4°C overnight. After washing, the membranes were incubated with horseradish peroxidase conjugated secondary antibody (Beyotime) for 45 min at 37°C. The membranes were then reacted with ECL reagent (Beyotime) and exposed to Fuji Rx 100 X-ray film (Fuji Photo Film, Tokyo, Japan) in darkness. The gray values of the blots were analyzed using Gel-Pro-Analyzer (Media Cybernetics, Bethesda, MD, USA). The following primary antibodies were used in the present study: Bcl-2, Bax, NF-κB p65, iNOS (Boster, Huhan, China), cleaved-caspase3, cleaved- poly ADP-ribose polymerase (PARP; Abcam), IκBα, p-p65^ser536^, Histone H3 (Bioss, Beijing, China), and β-actin (Santa Cruz Biotechnology, Inc., Dallas, TX, USA).

### Statistical Analysis

Data were represented at means ± SD. Statistical analysis was performed using SPSS 19.0 software (IBM, New York, NY, USA). Results of pathological scores were analyzed using Kruskal–Wallis test followed by Nemenyi test. Other results were analyzed using one-way analysis of variance (ANOVA) followed by Fisher’s least significant difference (LSD) test. A *P* value < 0.05 was considered statistical significance.

## Results

### Celastrol Alleviates EAE

**FIGURE 2:**
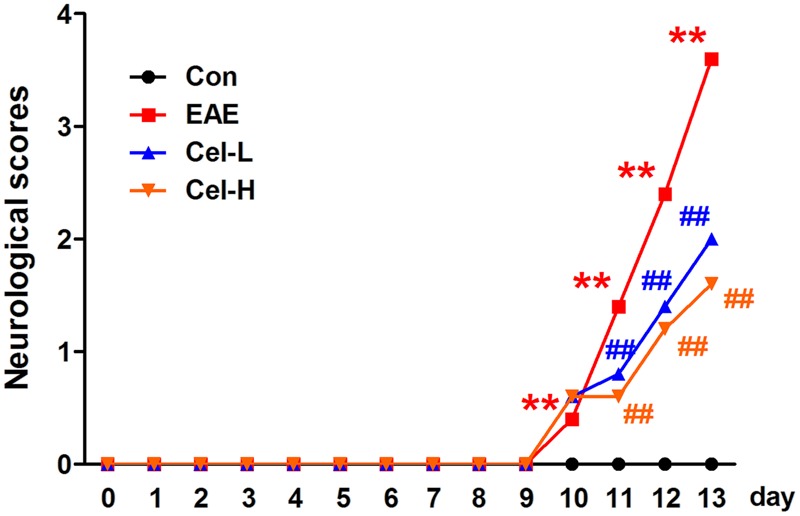
**Treatment of celastrol attenuates neurological severity of experimental autoimmune encephalomyelitis (EAE) rats.** Neither low nor high dose of celastrol delayed the onset of EAE, but both of the dosages decreased neurologic severity of EAE. Data were shown as mean ± SD. (*n* = 5). ^∗∗^*P* < 0.01 versus control group, ^##^*P* < 0.01 versus EAE group.

In the present study, the effects of celastrol on EAE complicated ON were evaluated on EAE rats. Low and high dosages of celastrol were intraperitoneally administrated immediately after establishment of the model. As illustrated in **Figure 2**, rats showed the first neurologic sign at day 10 (1.4 ± 0.5), which was similar to that obtained in previous studies (Samuvel et al., 2015). The neurologic score rapidly increased in the EAE group in the following days and reached 3.6 ± 0.5 at day 13. The scores in the two celastrol treated groups were also increased at day 10 but underwent markedly slower increasing rate in the following days. At day 13, the neurological score of rats treated with low dosage of celastrol was 2.0 ± 0.7, and the score in the high dosage group was 1.6 ± 0.5. Both of them were significantly lower than that in the EAE group (*P* < 0.01). This result indicates that although treatment of celastrol does not delay the onset of EAE, it dose-dependently attenuates EAE severity in rats.

### Celastrol Attenuated Inflammatory Infiltration and Demyelination in Spinal Cords of EAE Rats

**FIGURE 3:**
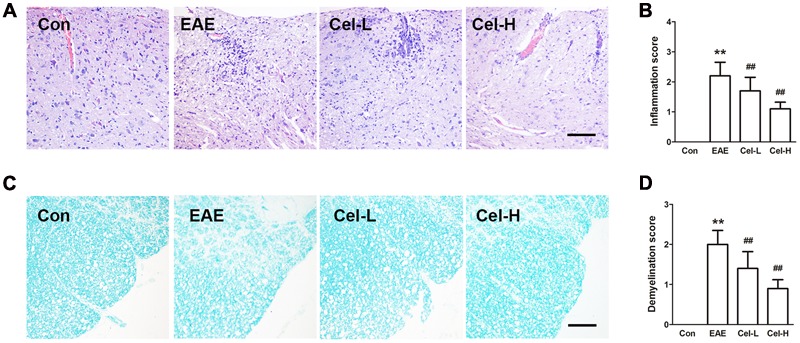
**Treatment of celastrol suppressed histopathological alterations and demyelination in spinal cords of EAE rats.** H & E **(A)** and Luxol fast blue (LFB; **C**) staining showed that celastrol attenuated inflammatory infiltration and demyelination in spinal cord of EAE rats, inflammatory **(B)** and demyelination **(D)** scores were significantly lowered in both low and high dosages of celastrol groups. Scale bars: 100 μm. Data were shown as mean ± SD. (*n* = 5). ^∗∗^*P* < 0.01 versus control group, ^##^*P* < 0.01 versus EAE group.

The rats were sacrificed on day 13 and the spinal cords were collected for histological examination. The results of H&E (**Figures 3A,B**) and LFB (**Figures 3C,D**) staining showed markedly inflammatory cells infiltration and demyelination in EAE rats. Treatment of celastrol significantly decreased the inflammatory infiltration and attenuated the demyelination. The pathological scores of high dosage of celastrol were lower than that of low dosage but did not reach significant.

### Celastrol Inhibited Production of Cytokines in Spinal Cords of EAE Rats

**FIGURE 4:**
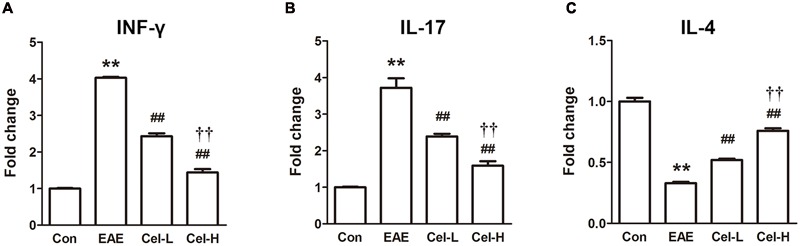
**Celastrol regulated production of cytokines and activation of NF-κB in spinal cords of EAE rats.** Celastrol dose-dependently downregulated the mRNA expression of INF-γ **(A)** and IL-17 **(B)** but upregulated IL-4 **(C)** in spinal cord of EAE rats. Data were shown as mean ± SD, *n* = 5. ^∗∗^*P* < 0.01 versus control group, ^##^*P* < 0.01 versus EAE group, ^††^*P* < 0.01 versus low dosage of celastrol group.

In EAE and MS, IFN-γ and IL-17 contribute to inflammation and myelin damage (El-Behi et al., 2011; Yan et al., 2012; Segal, 2014) and IL-4 is involved in the recovery of the disease (Payne et al., 2012). The mRNA expression of Th1/Th17 cytokines IFN-γ and IL-17 and Th2/Treg cytokines IL-4 was determined by quantitative real-time PCR. As shown in **Figure 4**, significant increases of IFN-γ and IL-17, and a significant decrease of IL-4 were observed in the EAE group compared to the control group (*P* < 0.05). Treatment of celastrol markedly downregulated the mRNA expression of IFN-γ and IL-17 and upregulated the mRNA expression of IL-4, with a more profound effect in the high dosage group compared to the low dosage group (*P* < 0.01).

### Celastrol Inhibited Production of Cytokines and Microgliosis in Optic Nerve of EAE Rats

**FIGURE 5:**
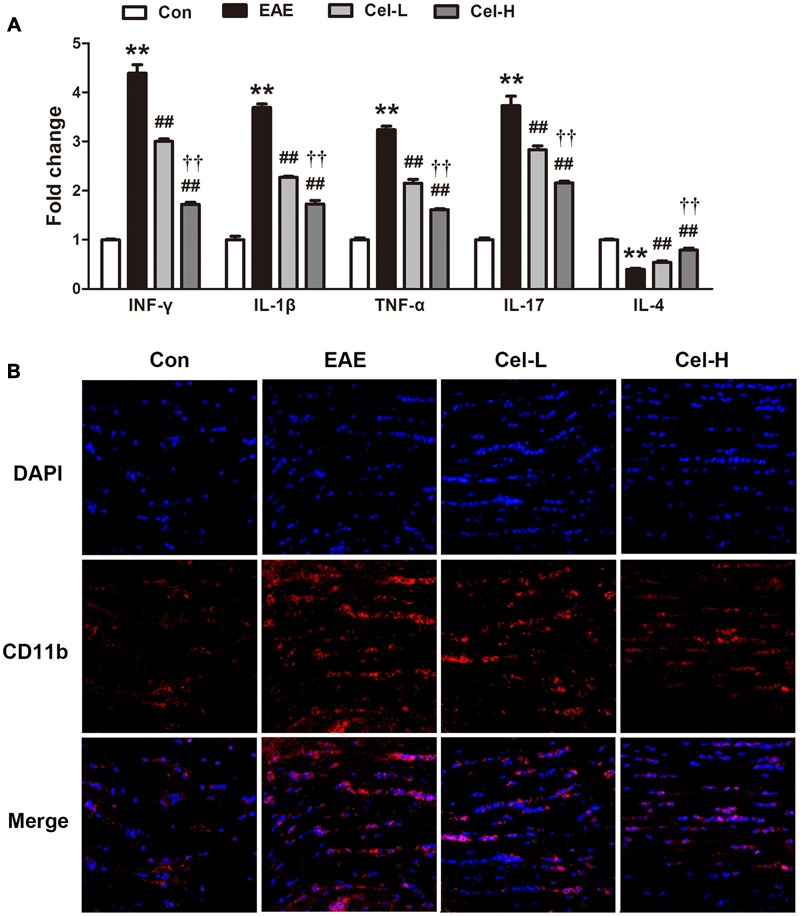
**Celastrol inhibited production of cytokines and microgliosis in optic nerve of EAE rats.** Celastrol dose-dependently downregulated the mRNA expression of INF-γ, IL-1β, TNF-α and IL-17 but upregulated IL-4 **(A)** in optic nerve of EAE rats. The expression of CD11b was decreased after treatment of celastrol **(B)**. Scale bar: 50 μm. Data were shown as mean ± SD, *n* = 5. ^∗∗^*P* < 0.01 versus control group, ^##^*P* < 0.01 versus EAE group, ^††^*P* < 0.01 versus low dosage of celastrol group.

In the optic nerve of EAE rats, we found the markedly upregulated mRNA expression of Th1/Th17 cytokines INF-γ, IL-1β, TNF-α and IL-17 (**Figure 5A**) and downregulated expression of Th2 cytokine IL-4. In line with the observations in the spinal cord, treatment of celastrol significantly diminished Th1/Th17 cytokines expression and enhanced IL-4 expression. The efficacy of high dosage was better than low dosage of celastrol (*P* < 0.01).

The effect of celastrol on microgliosis in optic nerve was evaluated as well. The optic nerve sections were immunofluorescence-stained with CD11b, a biomarker of activated microglia (**Figure 5B**). Acute EAE rats showed a notable increase of CD11b-positive areas, which indicated the occurrence of microgliosis. In contrast, treatment of celastrol exhibited markedly reduced microgliosis compared with EAE rats.

### Celastrol Inhibited Expression of iNOS and Activation of NF-κB in Optic Nerve of EAE Rats

**FIGURE 6:**
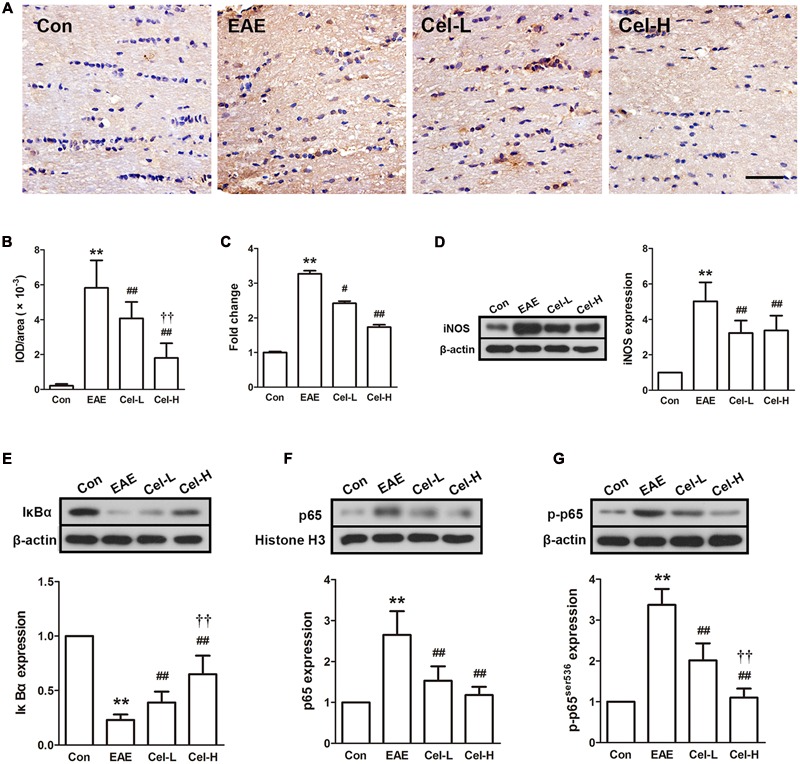
**Celastrol inhibited inducible nitric oxide synthase (iNOS) expression and activation of NF-κB in optic nerve in EAE rats. (A)** IHC staining of iNOS in optic nerve. **(B)** Quantification of iNOS-positive areas. **(C)** Quantitative real-time PCR analysis of iNOS expression. **(D)** Western blot analysis of iNOS expression. **(E–G)** Western blot analysis of IκBα, p65 and p-p65 expression, respectively. Treatment of celastrol reduced expression of iNOS and inhibited the activation of NF-κB in optic nerve in EAE rats. Scale bar: 100 μm. Data were shown as mean ± SD, *n* = 5. ^∗∗^*P* < 0.01 versus control group, ^##^*P* < 0.01 versus EAE group, ^††^*P* < 0.01 versus low dosage of celastrol group.

The expression of inflammation-associated protein iNOS, the enzyme responsible for nitric oxide production, and the changes of proteins in NF-κB signaling pathways were investigated in EAE and celastrol treated rats. The mRNA and protein expression levels of iNOS were dramatically enhanced in EAE rats, and were markedly decreased by celastrol (**Figures 6A–D**). Immune-staining of iNOS showed the similar pattern with that in Western blot analysis. NF-κB plays a crucial role in the inflammatory process. As a transcription factor, NF-κB leads to production of numerous inflammatory cytokines when it is activated. Therefore, it has become a potential therapeutic target for some inflammatory diseases. In the present study, we investigated the changes in levels of IκBα, phosphor-NF-κB p65 and nuclear NF-κB p65 in the optic nerve (**Figures 6E–G**). The results of Western blot analysis demonstrated that IκBα was significantly downregulated and the expression levels of p-p65 and nuclear p65 were upregulated in the optic nerve of EAE group compared to normal control group. Treatment of celastrol restored these changes in a dose-dependent manner.

### Celastrol Attenuates Ganglion Cells Apoptosis in the Retina of EAE Rats

**FIGURE 7:**
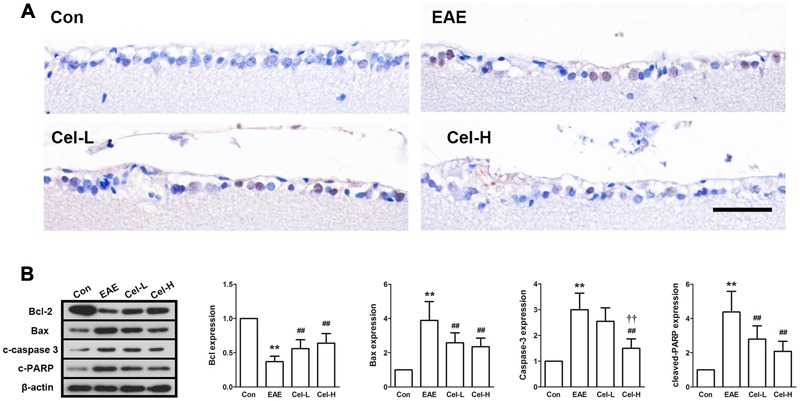
**Celastrol attenuates ganglion cells apoptosis in the retina of EAE rats.** Treatment of celastrol decreased the number of TUNEL-positive cells **(A)**, upregulated expression of Bcl-2 **(B)** and downregulated expression of Bax, cleaved-caspase 3 and cleaved-PARP. Scale bar: 100 μm. Data were shown as mean ± SD, *n* = 5. ^∗∗^*P* < 0.01 versus control group, ^##^*P* < 0.01 versus EAE group, ^††^*P* < 0.01 versus low dosage of celastrol group.

We then sought to determine whether ganglion cell death was inhibited in the retina of acute EAE animals after treatment with celastrol. Results of TUNEL staining demonstrated that in the RGC layer, numerous TUNEL-positive cells were observed in EAE rats. The number of TUNEL-positive RGC was notably decreased in the low-dose celastrol group compared to the EAE group. Few TUNEL-positive RGC was found in the high-dose celastrol group (**Figure 7A**). Mitochondria-mediated apoptotic pathway was also investigated in optic nerves. Results of Western blot analysis revealed that the expression of Bcl-2 was downregulated and the expression of Bax was upregulated in optic nerve of EAE rats. These changes indicated that the ratio of Bcl-2/Bax was made a pro-apoptotic shift. Meanwhile, increased expression of cleaved-caspase-3 and cleaved-PARP was also observed in EAE rats (**Figure 7B**). In consistence with the observation in TUNEL analysis, treatment EAE rats with celastrol markedly restored these changes, indicating that celastrol treatment protected RGC from acute EAE.

## Discussion

Multiple sclerosis is an autoimmune inflammatory demyelinating disease of the CNS without effective treatment. Optic nerve injury occurs early in the development progress of MS (Hobom et al., 2004). Celastrol is a natural compound that generally used for anti-inflammation and anti-autoimmunity. Previous studies have demonstrated that celastrol ameliorated EAE development by suppressing pathogenic Th17 responses and inhibiting inflammatory responses in spinal cord and brain (Abdin and Hasby, 2014; Wang et al., 2015). The present study designed to evaluate the effects of celastrol on MS and ON. Using MPB-induced EAE rats, we found that celastrol attenuated demyelination, inhibited production of pro-inflammatory cytokines and increased production of anti-inflammatory cytokines in spinal cord. In addition, we found the inflammatory response was also suppressed by celastrol in optic nerve, as well as the apoptosis of RGC in retina. These results indicate that celastrol alleviates the neurologic severity of EAE via suppressing demyelination and inflammation in spinal cord, and attenuates ON by inhibiting inflammation in optic nerve and preventing RGC apoptosis in retina.

Spinal cord injury is the primary cause of long-term disability in MS. Results of magnetic resonance imaging (MRI) detection show abnormalities in spinal cord in approximate 75% of the MS patients, and the cervical cord is the most common lesion site (Kidd et al., 1993; Nijeholt et al., 1998; Lukas et al., 2013). In addition, a previous MRI study demonstrated that early spinal cord lesions were predictive in disability in isolated ON patients converting to MS (Swanton et al., 2009). Inflammation in the CNS is considered to contribute to the spinal cord injury including demyelination, axon loss and atrophy in MS and EAE (Siffrin et al., 2010; Friese et al., 2014). In the present study, from the results of LFB staining, we found that celastrol significantly attenuated the demyelination in the spinal cord of EAE rats, which was consistent with its promoting effects in neurological function test. Meanwhile, H&E staining illustrated the reduced inflammatory infiltration in the spinal cord of celastrol-treated EAE rats. Based on the accepted links between disability, spinal cord injury and inflammation, we deduced that celastrol could attenuate neurological dysfunction in EAE rats, which might be associated with its effects on inhibiting demyelination and inflammation in the spinal cord.

Multiple sclerosis is an autoimmune disorder of the CNS and the infiltration of immune cells into the CNS occurs at the onset of the disease. Although detailed pathogenesis of MS is not well revealed, it is widely accepted that CD4^+^ T cells play important roles in this disease. CD4^+^ T cells are generally classified into helper-T-cell (Th)1, Th2, Th17 and regulatory T cell (Treg) according to the cytokines they secreted (Wan and Flavell, 2009). INF-γ and IL-17, which are secreted by Th1 and Th17, respectively, are considered as pro-inflammatory cytokines. In contrast, IL-4 and IL-10, which are produced by Th2 and Treg, are considered as anti-inflammatory cytokines. In normal condition, the activities of these cells of opposite functions reach a balance. In MS, the balance is broken, the pro-inflammatory cytokines are over-produced and the secretion of anti-inflammatory cytokines is suppressed, which induces the abnormal immune attack into the CNS (Benveniste et al., 2014; Segal, 2014). In EAE models, T cells are activated by myelin and migrate to the CNS, which mimics the immune cells infiltration as in MS. In the present study, the mRNA expression of INF-γ, IL-17 was downregulated and the expression of IL-4 was upregulated in the spinal cord of celastrol-treated EAE rats. This finding is consistent with the previous study in which celastrol was found to reduced the percentage of CD4^+^IL-17^+^ T cells in spinal cord (Wang et al., 2015), and indicates that celastrol attenuates spinal cord injury in EAE through re-balancing the pro-inflammatory Th1/17 and anti-inflammatory Th2/Treg factors.

Immune responses also play important roles in ON pathogenesis (Smith et al., 2011; Das et al., 2013). As introduced above, Th1/Th17 and Th2/Treg cells are also implicated in the pathological process of ON. In the current investigation, we determined the mRNA expression of INF-γ, TNF-α and IL-1β, which are produced by Th1, IL-17, which is produced by Th17, and IL-4, which is produced by Th2. In line with our observation in the spinal cord, dramatic increases in Th1 and Th17 cytokines and decrease in Th2 cytokine were found in the optic nerve in EAE rats. Treatment of celastrol markedly reversed this change so that the expression of Th1 and Th17 cytokines was downregulated, while IL-4 was upregulated. We and others have proved the effects of celastrol on immune cells regulation, and the present result extends the knowledge to optic nerve. In the CNS, gliosis, especially microgliosis, which is induced by the overproduced cytokines and other factors, is a hallmark of inflammation. Microglia is considered to be the immune system in the CNS. Microglia activation is a primary factor in the CNS against various injuries including inflammation (Kreutzberg, 1996). Our current data show that celastrol attenuates microgliosis in the optic nerve of EAE rats.

NF-κB is an important nuclear factor that regulates transcription of numerous inflammatory and immune-associated factors. Inhibition of NF-κB has been demonstrated to beneficial for treating ON in EAE (Das et al., 2013). Celastrol is a potent natural NF-κB inhibitor (Lee et al., 2006; Nam, 2006) and has been demonstrated to block NF-κB activation in various diseases including MS (Wang et al., 2015). The goal of this study was to extend the understanding of the NF-κB inhibitory effect of celastrol to optic nerve, and to examine the expression of NF-κB-regulated inflammatory-associated protein iNOS in EAE rats. Improper upregulation of iNOS results overproduction of NO, and the latter may contribute to the cell damage and myelin loss (Iwahashi et al., 1999; Danilov et al., 2003). We found that celastrol effectively inhibited NF-κB phosphorylation and nuclear translocation, and downregulated iNOS expression in optic nerve. Our results indicate that the anti-inflammatory effect of celastrol may contribute to its neuroprotective effects on optic nerve.

Axons damage is a primary pathological feature of MS and is the major cause of visual loss in this disease. Apoptosis of RGCs, the neurons that form the axons of optic nerve, has been found to occur early in the development of the disease in animal models of EAE (Meyer et al., 2001; Hobom et al., 2004). In the current study, retina of EAE rats showed obviously increased apoptotic RGCs, and treatment of celastrol markedly reduced apoptotic RGCs. In previous studies, apoptosis of RGCs has been found both before and after optic nerve inflammation in EAE, which indicates that inflammation is not the only reason of RGC apoptosis (Hobom et al., 2004; Shindler et al., 2006). We did not investigate the apoptosis in the very early time point after MBP immunization, which was a limitation of the present study. Therefore, inflammation may not the only reason that responsible for the apoptosis of RGCs in our study. In spite of this, the anti-inflammatory effect of celastrol may still, at least partly, contribute to the inhibition of RGCs apoptosis. In addition, mitochondrial apoptotic pathway, which was regulated by Bcl-2 family proteins, was also investigated in this study. It is well accepted that Bcl-2 is an anti-apoptotic protein and Bax is a pro-apoptotic protein. Generally, the ratio of Bcl-2/Bax is used to reflect the apoptotic tendency of target organs (Ola et al., 2011). In our study, treatment of celastrol resulted in upregulated Bcl-2 and downregulated Bax, which shifted the relation of these two proteins toward a more pro-survival ratio in RGCs. Meanwhile, cleaved-caspase 3 and PARP, two important proteins that mediate apoptosis, were downregulated by celastrol as well. Our study suggests that celastrol exerts an inhibitory effect on RGCs apoptosis in EAE rats. This effect may come from both the indirect effect of anti-inflammation and the direct effect of apoptotic-associated proteins regulation of celastrol.

Collectively, the present study confirms the neuroprotective effects of celastrol on EAE models through T cells activities regulation. In addition, our data indicate that celastrol attenuates ON in EAE via anti-inflammatory and anti-apoptotic effects. These findings provide new pre-clinical evidence for the use of celastrol in treatment of multiple sclerosis.

## Author Contributions

HY: Literature Search, Data Collection, Statistical Analysis, and Manuscript Preparation. CL, JJ, and YW: Data Collection. XZ: Study Design and Manuscript Preparation.

## Conflict of Interest Statement

The authors declare that the research was conducted in the absence of any commercial or financial relationships that could be construed as a potential conflict of interest.
